# Efficacy of internet-delivered acceptance and commitment therapy for severe health anxiety: results from a randomized, controlled trial

**DOI:** 10.1017/S0033291720001312

**Published:** 2021-11

**Authors:** Ditte Hoffmann, Charlotte Ulrikka Rask, Erik Hedman-Lagerlöf, Jens Søndergaard Jensen, Lisbeth Frostholm

**Affiliations:** 1 The Research Clinic for Functional Disorders and Psychosomatics, Aarhus University Hospital, Noerrebrogade 44, bldg. 2C, 1, 8000 Aarhus C, Denmark; 2 Department of Child and Adolescent Psychiatry, Aarhus University Hospital, Palle Juul-Jensens Boulevard 175, ent. K, 8200 Aarhus, Denmark; 3 Department of Clinical Medicine, Aarhus University, Palle Juul-Jensens Blvd. 82, 8200, Aarhus, Denmark; 4 Department of Clinical Neuroscience, Karolinska Institute, Tomtebodavägen 18A, 5, 171 77 Stockholm, Sweden

**Keywords:** Acceptance and commitment therapy, digital health, health anxiety, hypochondriasis, illness anxiety disorder, internet intervention, randomized controlled trial

## Abstract

**Background:**

Health anxiety is common, disabling and costly due to patients’ extensive use of health care services. Internet-delivered treatment may overcome barriers of accessibility to specialized treatment. We aimed to evaluate the efficacy of internet-delivered acceptance and commitment therapy (iACT).

**Methods:**

A randomized, controlled trial of iACT versus an internet-delivered discussion forum (iFORUM), performed in a Danish university hospital setting. Patients self-referred and underwent video-diagnostic assessment. Eligible patients (≥18 years) with health anxiety were randomized to 12 weeks of intervention. The randomization was blinded for the assessor. The primary outcome was between-group unadjusted mean differences in health anxiety symptoms measured by the Whiteley-7 Index (WI-7, range 0–100) from baseline to 6-month follow-up (6-MFU) using intention to treat and a linear mixed model. The study is registered at clinicaltrials.gov, number NCT02735434.

**Results:**

A total of 151 patients self-referred, and 101 patients were randomized to iACT (*n* = 53) or iFORUM (*n* = 48). A mean difference in change over time of 19.0 points [95% confidence interval (CI) 10.8–27.2, *p* < 0.001] was shown on the WI-7, and a large standardized effect size of *d* = 0.80 (95% CI 0.38–1.23) at 6-MFU. The number needed to treat was 2.8 (95% CI 1.8–6.1, *p* < 0.001), and twice as many patients in iACT were no longer clinical cases (35% *v.* 16%; risk ratio 2.17, 95% CI 1.00–4.70, *p* = 0.050). Adverse events were few and insignificant.

**Conclusions:**

iACT for health anxiety led to sustained effects at 6-MFU. The study contributes to the development of easily accessible treatment options and deserves wider application.

## Introduction

Health anxiety, often designated as hypochondriasis, is a global health problem on the rise affecting 1–5% of the adult population (Fink et al., 2004; Kosic, Lindholm, Jarvholm, Hedman-Lagerlof, & Axelsson, 2020; Sunderland, Newby, & Andrews, 2013) and is very common among medical patients (Weck, Richtberg, & Neng, 2014). It is characterized by obsessive rumination with fears of harboring a serious illness and tends to persist despite medical reassurance (Fink et al., 2004). Although patients frequently attend medical care, their primary disorder is rarely recognized and thus remains untreated. Consequently, health anxiety is associated with extensive use of health care services (Fink, Ornbol, & Christensen, 2010), increased rates of occupational disability, depression and anxiety, and lowered quality of life (Sunderland et al., 2013). These costs are potentially avoidable if effective treatment is available.

Fortunately, long-term cost-effective treatments do exist (Axelsson & Hedman-Lagerlof, 2019; Thomson & Page, 2007); so far, cognitive behavioral therapy (CBT) being the most frequently investigated. However, acceptance and commitment therapy (ACT), has also demonstrated promising results across a wide range of mental health problems (A-Tjak et al., 2015; Frostholm & Rask, 2019), including health anxiety (Eilenberg, Fink, Jensen, Rief, & Frostholm, 2016; Eilenberg, Hoffmann, Jensen, & Frostholm, 2017). ACT is considered a ‘third wave’ CBT approach that aims to increase patients' behavioral repertoire, also designated as ‘psychological flexibility’, in two ways: firstly by changing the way patients relate to distressing symptoms by gradually increasing the ability to ‘open up’ and ‘stay present’ to thoughts, feelings, and bodily sensations, and secondly to enhance engagement with value-based activities to create a meaningful life based on long-term goals and values (Hayes, 2016). In spite of evidence-based treatments, the limited availability of clinics or specialists and regional variability, restrict patients' access to treatment. Therefore, low intensity and easily accessible treatment options are needed (Holmes et al., 2018).

Harnessing new technologies has the potential to greatly increase the availability of evidence-based treatments (Holmes et al., 2018). One of these developments is internet-delivered psychological treatment programs, which consist of highly structured online modules accompanied by homework assignments and often clinician guidance. The common principle is that the treatment mirrors the same techniques and processes of change as in traditional face-to-face treatment. Internet-delivered treatment has several advantages such as independence of geographical distance to the clinic, fewer or no scheduled appointments, less interference with patients' daily life, and possibly less perceived stigma (Brown, Glendenning, Hoon, & John, 2016; Rusch, Angermeyer, & Corrigan, 2005). A recent meta-analysis found that internet-delivered CBT (iCBT) produced equivalent effects to face-to-face treatment for anxiety and mood disorders (Carlbring, Andersson, Cuijpers, Riper, & Hedman-Lagerlof, 2018). Four RCTs from two research groups have already shown that iCBT was effective, long-lasting, and cost-effective for health anxiety (Hedman, Axelsson, Andersson, Lekander, & Ljotsson, 2016; Hedman et al., 2011, 2013, 2014; Newby et al., 2018). However, importantly, internet-delivered ACT (iACT) seems to compare favorably to iCBT in terms of mean adherence to protocol (Brown et al., 2016), which is a well-documented limitation restraining the potential effectiveness of internet-delivered treatments (Donkin et al., 2011). In a recent pilot study, we showed that iACT was a feasible and potentially efficacious treatment for health anxiety (Hoffmann, Rask, Hedman-Lagerlof, Ljótsson, & Frostholm, 2018). Still, the efficacy of iACT for health anxiety needs to be investigated in a randomized design.

Although one of the key goals when developing a new treatment is to avoid harm, the potential adverse events are often neglected when examining psychological treatments. A meta-analysis of individual treatment responses found that symptom deterioration was frequently reported in iCBT (Rozental, Magnusson, Boettcher, Andersson, & Carlbring, 2017) underlining the need for additional systematic assessment of adverse events.

### The aim of the study

The aim of this study was to investigate the efficacy of iACT for health anxiety in an RCT. Based on our previous pilot study (Hoffmann et al., 2018), we expected that iACT would lead to clinically significant reductions of health anxiety, somatic symptoms, symptoms of anxiety and depression as well as increases in quality of life and psychological flexibility compared with an active control condition receiving an internet-delivered discussion forum (iFORUM). In addition, adverse events such as symptom deterioration and negative effects attributed to treatment were systematically examined.

## Methods

### Study design and participants

This study is an individually randomized, controlled trial of iACT *v.* an active control condition encompassing iFORUM, performed at the Research Clinic for Functional Disorders and Psychosomatics at Aarhus University Hospital, Denmark. Patients self-referred through the clinic's webpage using their unique national identification number to log into a portal consisting of; (1) written consent allowing the clinician to access the patient's health information in the national Electronic Patient Record prior to assessment, (2) subjective description of their health problem by enquiring ‘Please describe your health anxiety in your own words and how you feel at the moment?’, and (3) baseline questionnaires measuring health anxiety symptoms among other measures of mental and physical health. Information about this study was available on the clinic's webpage (http://funktionellelidelser.dk) and on the Danish anxiety association's webpage (www.angstforeningen.dk), and electronic information about the trial was also sent to general practitioners (GP) nationwide. To further facilitate recruitment, two patient videos were produced to illustrate symptoms of health anxiety and the principles of internet-delivered treatment. These were available on the webpage and shared through the hospital's Facebook page.

Eligible patients had severe health anxiety according to the empirically based diagnostic criteria established by Fink et al. (2004) and a self-reported Whiteley Index-7 (WI-7) score of >21.4 (scale range 0–100), which is established as a clinically relevant cut-off score (Fink et al., 2010). Health anxiety had to be the principal diagnosis if comorbid disorders were present according to the diagnostic assessment based on the 10th edition of the International Classification of Diseases and Related Health Problems (ICD-10) (World Health Organization, Collaborating Centres for Classification of Diseases, 2014). Patients were ⩾18 years and able to speak, read, and write Danish, were Danish residents, and had access to computer and internet. Exclusion criteria were acute suicidal risk, current abuse of narcotics, alcohol, or non-prescribed medication, life-time diagnosis of psychoses, bipolar affective disorder or depression with psychotic symptoms (ICD-10: F20–29, F30–31, F32.3, F33.3), and pregnancy at time of trial entry. Other exclusion criteria were former treatment for health anxiety at the clinic, lack of informed consent, and changes in anxiety medication within the past 2 months. If patients had changed dose or recently started anxiety medication, they were preliminarily included and reassessed for eligibility after 2 months of stable medication. Patients were asked to keep a stable dosage throughout the trial.

### Assessment

Self-referrals were initially screened by a psychologist (first author, DH) in accordance with the eligibility criteria, and ineligible patients were telephoned and advised to consult their GP. Potentially eligible patients were invited to a thorough video-based clinical assessment. Health information from referral questionnaires and the electronic patient records were examined prior to the assessment. Trained psychologists and a psychiatrist conducted a shortened and modified version of the diagnostic interview Schedules for Clinical Assessment in Neuropsychiatry (SCAN) (Petersen et al., 2019) assessing health anxiety (Fink et al., 2004) and the corresponding diagnoses of hypochondriacal disorder according to ICD-10 (World Health Organization, Collaborating Centres for Classification of Diseases, 2014) as well as illness anxiety disorder (IAD) and somatic symptom disorder (SSD) according to DSM-5, respectively (American Psychiatric Association, 2013). The interview also screened for major depressive disorder, anxiety disorders, obsessive-compulsive disorder, and somatoform disorders based on the criteria from ICD-10. The assessment lasted 1–2 hours including a brief patient history, a clinical summary, and information about the project. Subsequently, eligible patients had 2 weeks to provide written consent and complete the randomization through the web portal. This allowed time for patients to consider whether they wanted to proceed to the final inclusion and randomization.

With consent, assessments were video recorded, and assessors rated and discussed clinical cases in supervision. The treatment, as well as technical issues, were also discussed at the weekly supervisions. An external supervisor with extensive experience with internet-delivered treatment and health anxiety (EHL) participated monthly. Medical supervision was provided by a psychiatrist (CUR) and by medical doctors at the clinic when needed.

### Randomization and masking

Eligible patients were randomly assigned to receive either iACT or iFORUM in a 1:1 computer-generated ratio with no restrictions or matching allowing up to 150 patients in total. After clinical assessment, the randomization followed automatically the next time the patient logged in, i.e. the assessor was blinded to the forthcoming allocation. Randomized patients were allocated to the next available therapist; yet pairing patients to the initial assessor when possible.

### Intervention

The iACT program was based on an existing, empirically supported manual for group-based ACT (ACT-G) (Eilenberg et al., 2016). The treatment platform was developed as a web app with a responsive design allowing access through computers, mobile devices, and tablets. The development, content, and feasibility of iACT have been presented elsewhere (Hoffmann et al., 2018). In brief, iACT was a clinician-guided, self-help program consisting of seven modules opened consecutively over 12 weeks of treatment (see online Supplementary material online for a ‘Program demonstration’). Four psychologists and a trainee psychology student provided the written clinical guidance, which was not restricted by predetermined templates. Messages were answered within 48 h on weekdays. The modules featured fixed content such as text, illustrations, audio files (mindfulness exercises), video clips, and interactive worksheets automatically stored and shared with the clinician, and an encrypted and embedded message system enabling written communication. An automatic (mobile) text message system notified patients about new modules, messages, questionnaires, or low activity. Likewise, a clinician-monitored control panel notified clinicians about patients' activity (e.g. missed questionnaires).

The iFORUM consisted of seven discussion forums consecutively opened over 12 weeks with a new topic related to health anxiety such as health care, relationships, or work. Patients were encouraged to share their experiences anonymously. The discussions were monitored for ethical reasons, but aside from that, there was no clinician interference. Thus, iFORUM aimed to control for the effect of emotional support and having contact to the health care system and can therefore not be considered as an active specific treatment but as an active control condition with unspecific effects. After 6-month follow-up (6-MFU), the patients from iFORUM were invited to cross over to iACT.

### Outcomes

Self-report Questionnaires were administered at baseline, month 1 (randomization), 2 (4 weeks into treatment), 3 (8 weeks into treatment), 4 (post-treatment), and 10 months after baseline (i.e. 6-MFU) (see online Supplementary material online regarding ‘Questionnaires’). Patients were telephoned and asked to answer the primary outcome measure if data were missing at post-treatment or at 6-MFU.

The primary outcome was changes in self-reported health anxiety symptoms from baseline to 6-MFU measured by the 7-item WI-7 (Fink et al., 1999). Patients rated their illness worries during the last 4 weeks on a 5-point rating scale from 1 = ‘Not at all’ to 5 = ‘A lot’ (scale range: 7–35) in response to questions like: ‘Do you worry a lot about your health’. The WI-7 has been shown to have high reliability and good external validity (Christensen, Bech, & Fink, 2010). To further enable comparison with former trials, we included the Health Anxiety Inventory Short-form (SHAI) as a secondary outcome measuring 18 health anxiety items on a categorical 4-point scale from 1 to 4 (scale range: 18–72) (Salkovskis, Rimes, Warwick, & Clark, 2002). The SHAI has demonstrated high reliability, criterion validity, and sensitivity to treatment (Alberts, Hadjistavropoulos, Jones, & Sharpe, 2013).

Other secondary outcomes were symptoms of depression, anxiety, and somatic symptoms measured on subscales of the Symptom Checklist-92 (SCL-92) (Derogatis & Cleary, 1977), namely SCL-dep (13 items, scale range: 13–65), SCL-anx (10 items, scale range: 10–50), and SCL-som (12 items, scale range: 12–60). All items were rated on a 5-point rating scale ranging from 1 = ‘Not at all’ to 5 = ‘A lot’. Quality of life was measured on the 5-item WHO Well-being Index (WHO-5) (Topp, Ostergaard, Sondergaard, & Bech, 2015) on a 6-point rating scale ranging from 0 = ‘At no time’ to 5 = ‘All of the time’ (scale range: 0–25). Psychological flexibility was measured by the 7-item Acceptance and Action Questionnaire-II (AAQ-II) (Bond et al., 2011) employing a 7-point rating scale from 1 = “Never true” to 7 = ‘Always true’ (scale range: 7–49).

All scales were transformed into a 0–100 score point scale ((score-min)/(max-min)) × 100 to facilitate comparison of changes between measures in this study and previous ones (Eilenberg et al., 2016).

Adverse events encompassing symptom deterioration on the WI-7 from baseline to 6-MFU, events requiring acute hospitalization, and negative effects were summarized. Negative effects were measured at 4 months (post-treatment) by the 32-item Negative Effects Questionnaire (NEQ) measuring six factors encompassing symptoms, hopelessness, failure, stigma, dependency, and quality (Rozental, Kottorp, Boettcher, Andersson, & Carlbring, 2016). Each negative effect was attributed to either ‘The treatment I received’ or ‘Other circumstances’, and the impact was rated from 0 = ‘Not at all’ to 4 = ‘Extremely’.

Adherence was summarized as the median number of completed modules, and treatment completion was defined a priori as three or more modules completed (Hoffmann et al., 2018). Patient activity also encompassed number of logins, submitted worksheets, and messages sent. Treatment satisfaction was rated on a 6-point rating scale at post-treatment by the following questions; ‘Would you recommend the treatment program to others’, ‘What do you think about receiving psychological treatment over the internet’ and ‘How useful did you find the information in the internet program’?

### Statistical analysis

Based on data from a previous RCT of ACT-G from the clinic (Eilenberg et al., 2016), we expected a 17-point difference in improvement between-groups on the WI-7. Power calculations showed that 45 patients in each group would be required to yield 80% power to detect a difference of 17 points (0–100) with an alpha level of 0.05 and 25% data attrition.

Data were summarized using either the mean and standard deviation (s.d.) for normally distributed variables, the median and interquartile range (IQR) for skewed variables, or the count and percentage for categorical variables. The primary outcome (WI-7) and the secondary outcomes (SHAI, SCL-dep, SCL-anx, SCL-som, WHO-5 and AAQ-II) were all analyzed using a linear mixed model with the given outcome as the dependent variable and group × time as a categorical variable and their interaction as the only independent variables. Using this model, we first calculated the mean score of the outcome in both groups at each time point and then tested whether there was a significant interaction effect (i.e. different changes over time in the groups). If this was the case, the treatment effect was calculated as the mean difference in change over time from baseline to 6-MFU. The model was also used to calculate between-group effect sizes at all timepoints (Cohen's d), i.e. the difference between the mean scores of iACT and iFORUM at each time point divided by the pooled s.d., and within group effect sizes (standardized response mean, SRM), i.e. the difference between the mean scores at 6-MFU and baseline divided by the s.d. of the difference. Due to a baseline difference in age, we made a sensitivity analysis in which we adjusted for age in our linear mixed model for the WI-7.

Clinically significant improvement was calculated using the criteria proposed by Fallon et al. (2017), where patients had to have a double improvement of 25% or greater over baseline scores on two measures of health anxiety, namely the WI-7 and the SHAI in our trial. The proportion of patients in the two groups with a clinically significant improvement was compared using a risk ratio (RR), and an estimate was calculated of the number needed to treat (NNT) to achieve one additional case of clinically significant improvement. Furthermore, we calculated and compared the proportion of patients scoring below the cut-off for non-clinical cases on the WI-7 < 21.4 at 6-MFU using a RR (Fink et al., 2010).

Finally, negative effects were summarized based on the NEQ. We tested whether negative effects were associated with treatment completion, i.e. number of modules completed or symptom deterioration on WI-7 from baseline to 6-MFU by using Wilcoxon rank-sum tests. All analyses were done on an intention to treat (ITT) basis using Stata version 15.1 for Windows.

## Results

### Self-referral

**Fig. 1.**
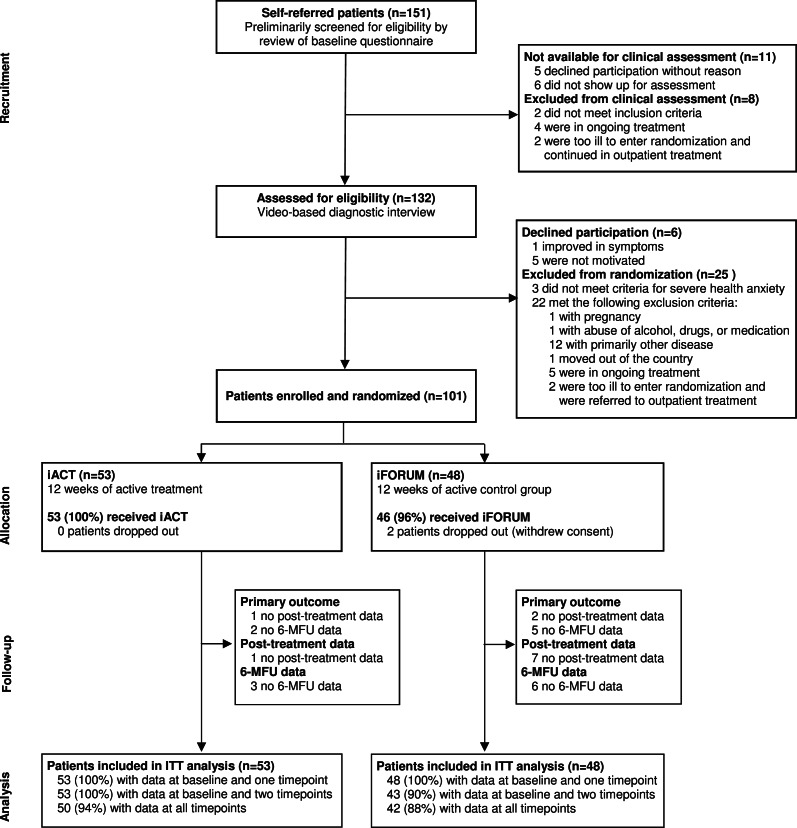
CONSORT Trial profile. iACT, internet-delivered Acceptance and Commitment Therapy.

Between 18 March 2016 and 29 March 2017, 151 adult patients self-referred to the trial (Fig. 1). Of the total, 38% found the treatment via the internet themselves, whereas the remaining patients learned about it from their GP (23%), a friend (15%), through advertisement (11%), or other sources (13%). In total, 132/151 (87%) were screened for eligibility, and 101/151 patients (66%) were included in the trial. The main reason for exclusion at assessment was another principal psychiatric disorder. Only six eligible patients declined participation. Compared to the final sample, they did not differ on demographic characteristics or baseline level of health anxiety [*t*(105) = 1.48, *p* *=* 0.14]. Eligible patients were randomly assigned to receive iACT (*n* = 53) or iFORUM (*n* = 48). Baseline demographics and clinical characteristics are summarized in Table 1.

**Table 1.** Baseline demographics and clinical characteristics

	iACT (n = 53)	iFORUM (n = 48)
**Demographic data**		
Age, mean years (SD) Age, min–max	37.2 (9.7) 19–61	42.3 (9.6) 20–63
Sex, n (%)		
Female	34 (64)	32 (67)
Male	19 (36)	16 (33)
Married or living with a partner, n (%)	42 (79)	36 (75)
Education, n (%)		
Unskilled	6 (11)	4 (8)
Skilled	5 (9)	7 (15)
Higher education (<4 years)	28 (53)	22 (46)
Higher education (>4 years)	11 (21)	14 (29)
Other	3 (6)	1 (2)
Work status, n (%)		
Employed or student	36 (68)	37 (77)
Unemployed	6 (11)	3 (6)
Disability pension or flexible work	3 (6)	5 (10)
Other (e.g. maternity leave)	8 (15)	3 (6)
Sick leave, n (%)		
Full-time sick leave	4 (8)	3 (6)
Part-time sick leave	7 (13)	4 (8)
No absence	35 (66)	38 (79)
Not working	7 (13)	3 (6)
**Clinical data**		
Health anxiety		
WI-7 health anxiety, mean (SD)^a^	75.5 (14.6)	74.3 (16.5)
Illness onset, mean age (SD)	21.6 (9.5)	26.1 (14.0)
Illness duration, median (IQR)^b^	12.8 (6.0 - 25.5)	11.7 (5.4 - 25.0)
Care-seeking type, n(%)^c^	43 (81)	37 (77)
Care-avoidant type, n(%)^c^	9 (17)	11 (23)
Psychiatric co-morbidity (ICD-10), n(%)		
Anxiety disorders^d^	12 (23)	12 (25)
Depressive disorder (mild to moderate)^e^	8 (15)	13 (27)
OCD^f^	1 (2)	2 (4)
Somatoform disorders^g^	19 (36)	11 (23)
At least one of the above diagnoses	31 (58)	28 (58)
Health anxiety classification (DSM-5), n(%)		
Illness anxiety disorder	25 (47)	28 (58)
Somatic symptom disorder	26 (49)	15 (31)
Use of antidepressants at assessment, n(%)	11 (21)	8 (17)

Data are number(%), mean(SD) or median(IQR). iACT = internet-delivered Acceptance and Commitment Therapy. iFORUM = internet-delivered discussion forum. n = Number of participants. SD = Standard deviation. WI-7 = Whiteley-7 Index. IQR = interquartile range. SCL = Symptom Checklist. WHO = World Health Organization. ICD = International statistical classification of diseases and related health problems. DSM = Diagnostic and Statistical Manual of Mental Disorders. OCD = Obsessive-compulsive disorder.

a Scale range 0-100 points. Higher scores indicate more symptoms, except for quality of life and psychological flexibility, where a high score indicate better functioning.

b Median and IQR reported due to a skewed distribution.

c Care-seeking and care-avoidant type is a classification pertaining to DSM-5 Illness anxiety disorder.

d ICD-10 (F40-41.9).

e ICD-10 (F32.0-32.1).

f ICD-10 (F42.0-42.1).

g ICD-10 (F45.0-45.1).

### Attrition and adherence

There was no data loss at baseline and at randomization (Fig. 1). Full primary outcome data at all time points were available for 90/101 (89%) patients, and partial data including both baseline and 6-MFU were available for 94/101 (93%) patients. Two patients withdrew their consent but were still included in the ITT.

In terms of treatment adherence, the median number of completed modules was 7 (IQR 5–7) in iACT and 2 (IQR 0–7) in iFORUM (see online Supplementary material online regarding ‘Treatment adherence’). No patients receiving iACT completed less than three modules which were previously defined as cut-off for treatment non-completion (Hoffmann et al., 2018). Patients in iACT sent a median of 14 (IQR 10–20) messages to their clinician, whereas the median number of postings among patients in iFORUM was 2.5 (IQR 0–6.5). The majority of patients in iFORUM (30/48 (63%)) posted at least one message.

### Primary outcome measure

**Fig. 2.**
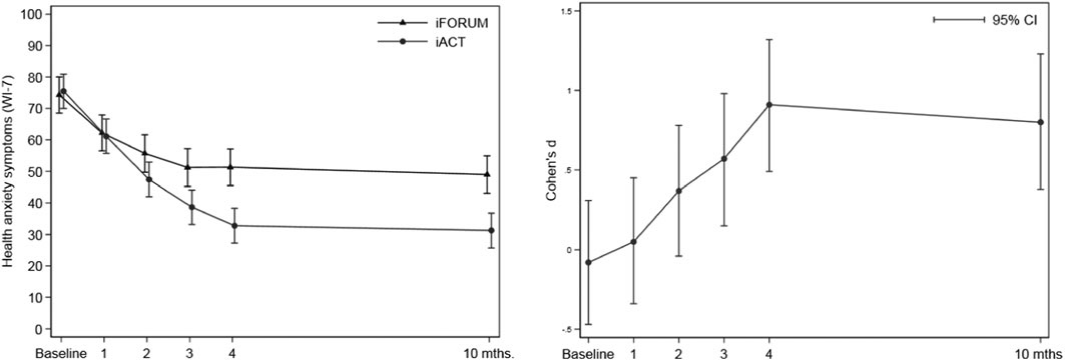
Effect of the treatment on the primary outcome: health anxiety symptoms. Effect of the treatment on the primary outcome WI-7 based on a linear mixed model. The left graph shows the mean values and 95% CI of two treatment groups at each time point (smaller values are in favor of the treatment). The right graph illustrates the unadjusted Cohen's d effect sizes with 95% CI at all time points throughout treatment. Positive effect sizes are in favor of the treatment. Baseline corresponds to the time of self-referral and 1 month to randomization and treatment initiation after the diagnostic assessment.

Figure 2 displays the improvement on the primary outcome measure, WI-7, and Table 2 presents the means, confidence interval (CI), and effect sizes at baseline and 6-MFU. There was different development over time in the two groups on WI-7 as indicated by the significant interaction effect between group and time [χ^2^(5) = 39.97, *p* < 0.001]. The unadjusted difference in mean improvement from baseline to 6-MFU was 19.0 points (95% CI 10.8–27.2) in favor of iACT. The standardized between-group effect size on WI-7 at 6-MFU was *d* *=* 0.80 (95% CI 0.38–1.23).

**Table 2.** Summary of results

Measures^a^	iACT (*n* = 53)				iFORUM (*n* = 48)				Between groups (*n* = 101)	
	Baseline (T1) Mean (95% CI)	6-MFU (T2) Mean (95% CI)	%^b^ change	SRM^c^ (95% CI)	Baseline (T1) Mean (95% CI)	6-MFU (T2) Mean (95% CI)	%^b^ change	SRM^c^ (95% CI)	6-MFU (T2) Unadjusted Cohens's *d*^d^ (95% CI)	6-MFU (T2) Unadjusted difference of improvement (95% CI)
Primary outcome										
Health anxiety (WI-7)	75.5	31.3	−58.5	2.16	74.3	49.0	−34.1	1.07	0.80	19.0**
	(70.0–80.9)	(25.7–36.8)		(1.65–2.67)	(68.5–80.0)	(43.1–54.9)		(0.7–1.45)	(0.38–1.23)	(10.8–27.2)
Secondary outcomes										
Illness worry (SHAI)	65.7	39.6	−39.7	1.70	66.9	55.3	−17.3	0.71	0.95	14.4**
	(61.9–69.4)	(35.8–43.5)		(1.27–2.14)	(63.0–70.9)	(51.2–59.5)		(0.38–1.05)	(0.52–1.39)	(8.6–20.2)
Depression (SCL-dep)	50.2	23.3	−53.6	1.30	44.0	32.0	−27.3	0.58	0.45	15.0**
	(44.9–55.6)	(17.8–8.7)		(0.92–1.68)	(38.4–49.6)	(26.2–37.9)		(0.26–0.91)	(0.04–0.86)	(7.3–22.7)
Anxiety (SCL-anx)	51.4	24.3	−52.7	1.58	48.2	30.1	−37.6	0.75	0.31	9.1*
	(46.2–56.7)	(18.9–29.6)		(1.16–2.00)	(42.7–53.7)	(24.4–35.9)		(0.41–1.09)	(−0.10 to 0.72)	(1.00–17.1)
Physical symptoms (SCL-som)	45.0	22.6	−49.8	1.26	37.8	28.9	−23.5	0.42	0.33	13.5**
	(40.0–50.0)	(17.5–27.8)		(0.89–1.63)	(32.5–43.0)	(23.4–34.4)		(1.11–0.73)	(−0.08 to 0.74)	(6.3 to –20.7)
Quality of life (WHO-5)	31.8	55.6	74.8	1.24	31.0	43.8	41.3	0.66	0.62	−11.0*
	(26.8–36.7)	(50.6–60.6)		(0.87–1.61)	(25.8–36.2)	(38.4–49.1)		(0.33–0.99)	(0.20–1.03)	(−17.5 to −4.6)
Psychological flexibility (AAQ-II)	38.4	61.3	59.6	1.11	41.8	51.8	23.9	0.57	0.47	−12.8**
	(33.4–43.5)	(56.2–66.4)		(0.76–1.46)	(36.5–47.1)	(46.3–57.3)		(0.25–0.90)	(0.06–0.88)	(−19.0 to −6.6)

iACT, internet-delivered Acceptance and Commitment Therapy; iFORUM, internet-delivered discussion forum; *n*, number of participants; CI, confidence interval; SRM, standardized response mean; WI-7, Whiteley-7 Index; SHAI, health anxiety inventory short-form; SCL, Symptom Checklist; WHO-5, World Health Organization-5 well-being index; AAQ, acceptance and action questionnaire.

All numbers in the table are calculated from the estimated mixed model.

a Scale range 0–100 points. Higher scores indicate more symptoms, except for quality of life and psychological flexibility, where a high score indicate better functioning.

b %-change = (T2/T1 × 100)–100.

c SRM: standardized response mean = (mean_T2 – mean_T1)/s.d.(T2–T1).

d Cohen's d was calculated as the difference between the mean scores of iACT and iFORUM at T2 divided by the pooled s.d. at T1.

*p < 0.05, **p < 0.001.

From baseline to 6-MFU, 34/50 (68%) patients in iACT had a clinically significant improvement of ⩾25% improvement over baseline on both the WI-7 and SHAI compared to 14/43 (33%) patients in iFORUM, corresponding to an RR of 2.09 (CI 95% 1.31–3.33, *p* = 0.002). The estimated NNT to achieve one additional case of clinically significant improvement with iACT compared to iFORUM was 2.8 (CI 95% 1.8–6.1, *p* *<* 0.001).

Furthermore, 18/51 (35%) patients in iACT had a WI-7 score <21.4 score point at 6-MFU and were no longer clinical cases compared to 7/43 (16%) patients in iFORUM which were a statistically significant between-group difference (RR 2.17; 95% CI 1.00–4.70, *p* *=* 0.050).

When adjusting for age, the treatment effect was of a similar magnitude as the unadjusted treatment effect, 19.0 points (95% CI 10.8–27.2).

### 3.4 Secondary outcome measures

All secondary outcomes revealed a significant interaction effect between group and time with *p* values ranging from <0.001 to 0.009. As seen in Table 2, the between-group effect sizes ranged from small to large at 6-MFU in favor of iACT (*d* *=* 0.31 to 0.95).

### 3.5 Adverse events

No patients experienced any serious adverse events requiring acute hospitalization, and only patients in iFORUM experienced symptom deterioration on the WI-7 (5/43 (11.6%)). At least one negative effect attributed to treatment was reported by 41/52 (78.8%) and 19/41 (46.3%) patients in iACT and iFORUM, respectively, with a mean number of 3.8 and 3.1 among the affected patients (Table 3). The item most frequently reported in iACT was; ‘I experienced more anxiety’ (item 3). There were few negative effects related to dependency of the treatment or clinician, feelings of failure, hopelessness or stigma. Negative effects were neither associated with treatment completion in iACT (*z* = 0.163, *p* *=* 0.87) and iFORUM (*z* = −1.326, *p* *=* 0.19), nor to symptom deterioration on WI-7 from baseline to 6-MFU in iACT [*t*(49) = 0.195, *p* *=* 0.85] and iFORUM [*t*(41) = −0.911, *p* *=* 0.37].

**Table 3.** Frequency, mean and standard deviation of self-reported negative effects

	iACT (*n* = 52)		iFORUM (*n* = 41)		Factor
	*n* (%)	Mean (s.d.)	*n* (%)	Mean (s.d.)	
Reporting any type of negative effect attributed to treatment	41 (78.8)	3.83 (2.87)	19 (46.3)	3.11 (2.18)	
Single items^a^					
3. I experienced more anxiety	19 (36.3)	1.89 (0.66)	1 (2.4)	2.00 (0.00)	Symptoms
13. Unpleasant memories resurfaced	17 (32.7)	1.47 (0.72)	1 (2.4)	1.00 (0.00)	Symptoms
11. I experienced more unpleasant feelings	16 (30.8)	1.75 (0.77)	0	0	Symptoms
4. I felt more worried	10 (19.2)	1.80 (0.92)	0	0	Symptoms
22. I did not always understand my treatment	9 (17.3)	0.78 (0.83)	9 (22.0)	1.11 (0.93)	Quality
2. I felt like I was under more stress	8 (15.4)	1.38 (0.52)	0	0	Symptoms
1. I had more problems with my sleep	7 (13.5)	2.00 (1.29)	0	0	Symptoms
12. I felt that the issue I was looking for help with got worse	7 (13.5)	2.00 (0.58)	2 (4.9)	2.50 (0.71)	Symptoms
27. I felt that my expectations for the treatment were not fulfilled	7 (13.5)	1.43 (0.79)	11 (26.8)	1.73 (0.90)	Quality
30. I felt that the treatment did not suit me	7 (13.5)	2.14 (0.90)	6 (14.6)	1.17 (0.75)	Quality
9. I felt sadder	6 (11.5)	1.50 (0.55)	0	0	Symptoms
26. I felt that the treatment did not produce any results	5 (9.6)	1.60 (0.55)	8 (19.5)	1.75 (0.89)	Quality
31. I felt that I did not form a closer relationship with my therapist	5 (9.6)	1.00 (1.00)	2 (4.9)	2.50 (0.71)	Quality
20. I think that I have developed a dependency on my treatment	5 (9.6)	0.80 (0.84)	1 (2.4)	0.00 (0.00)	Dependency
5. I felt more dejected	4 (7.7)	2.00 (0.00)	1 (2.4)	1.00 (0.00)	Symptoms
21. I think that I have developed a dependency on my therapist	3 (5.8)	1.00 (1.00)	0	0	Dependency
15. I got thoughts that it would be better if I did not exist anymore and that I should take my own life	2 (3.8)	1.50 (0.71)	0	0	Symptoms
7. I experienced lower self-esteem	2 (3.8)	1.50 (0.71)	0	0	Failure
8. I lost faith in myself	2 (3.8)	1.50 (0.71)	0	0	Failure
10. I felt less competent	2 (3.8)	1.00 (0.00)	0	0	Failure
6. I experienced more hopelessness	2 (3.8)	2.00 (0.00)	1 (2.4)	1.00 (0.00)	Hopelessness
18. I started thinking that the issue I was seeking help for could not be made any better	2 (3.8)	1.00 (0.00)	1 (2.4)	2.00 (0.00)	Hopelessness
19. I stopped thinking help was possible	0	0	0	0	Hopelessness
23. I did not always understand my therapist	2 (3.8)	1.00 (1.41)	0	0	Quality
24. I did not have confidence in my treatment	2 (3.8)	1.00 (0.00)	3 (7.3)	1.00 (1.73)	Quality
17. I stopped thinking that things could get better	2 (3.8)	1.00 (0.00)	1 (2.4)	2.00 (0.00)	Hopelessness
14. I became afraid that other people would find out about my treatment	1 (1.9)	2.00 (0.00)	2 (4.9)	1.50 (0.71)	Stigma
16. I started feeling ashamed in front of other people because I was having treatment	1 (1.9)	2.00 (0.00)	0	0	Stigma
28. I felt that my expectations for the therapist were not fulfilled	1 (1.9)	1.00 (0.00)	0	0	Quality
29. I felt that the quality of the treatment was poor	1 (1.9)	2.00 (0.00)	4 (9.8)	1.50 (1.90)	Quality
32. I felt that the treatment was not motivating	1 (1.9)	3.00 (0.00)	1 (2.4)	2.00 (0.00)	Quality
25. I did not have confidence in my therapist	0	0	0	0	Quality

iACT, internet-delivered Acceptance and Commitment Therapy; iFORUM, internet-delivered discussion forum; *n*, number of participants; s.d., standard deviation.

a Only negative effects attributed to treatment. The most frequent items are presented at the top.

### Additional help-seeking and treatment satisfaction

No statistically significant difference was found in additional help-seeking during treatment in both groups [χ^2^(1) = 0.71, *p* *=* 0.40]. Regarding patient satisfaction, 47/52 (90%) of the patients receiving iACT reported that they would ‘Definitely’ or ‘Most likely’ recommend the treatment program to others. Most patients, 46/52 (88%), found receiving treatment over the internet either ‘Very good’ or ‘Good’, and the information in iACT was found to be ‘Very useful’ or ‘Mostly useful’ to 49/52 (94%) patients in iACT.

## Discussion

### Main findings

We found that 12 weeks of clinician-guided iACT significantly decreased symptoms of health anxiety compared to an active control condition. Improvements were sustained at 6-MFU with an unadjusted mean difference of 19.0 points and a large standardized between-group effect size on the primary outcome. The NNT was 2.8 to achieve one case of clinically significant improvement, and 35% of patients in iACT were no longer clinical cases (Fink et al., 2010). In addition, patients receiving iACT also reported significant improvements on all secondary outcomes compared to patients in iFORUM, and no patients in iACT reported symptom deteriorations on the primary outcome at 6-MFU. The negative effects attributed to treatment were neither associated with treatment completion nor with the final outcome. Taken together, the results showed that clinician-guided iACT can be a highly effective and acceptable treatment for patients with health anxiety.

### Comparison to other studies

Since this was the first RCT to investigate iACT for health anxiety, our results cannot be directly compared to studies using the same treatment model and type of delivery. However, one RCT investigated group-based ACT (ACT-G) for health anxiety and found an unadjusted mean difference on the WI-7 of 20.5 points (95% CI 11.7–29.4) from baseline to 6-MFU (Eilenberg et al., 2016), which is almost equivalent to the effect of iACT. Moreover, 14/52(27%) patients attending ACT-G were no longer clinical cases as judged by the cut-off score on WI-7 compared to 35% in iACT thus suggesting comparable efficacy of face-to-face and internet-delivered ACT for health anxiety. The content of the iACT program was based on the ACT-G manual, which supports the notion that internet-delivered treatment is a new and feasible way to deliver the same therapeutic principles.

Our results were also in accordance with the four previous trials on iCBT for health anxiety (Hedman et al., 2011; 2014), IAD and SSD (Hedman et al., 2016; Newby et al., 2018). Even though comparison is hampered by the various outcome measures applied, all studies with a control condition found large between-group effect sizes on their primary outcome (Hedman et al., 2011, 2016; Newby et al., 2018).

In terms of adherence, iACT is said to compare favorably to iCBT (Brown et al., 2016). A meta-analysis found that patients in iCBT in average completed 81% of their treatment (van Ballegooijen et al., 2014). In our study, patients averagely completed 88% of their treatment modules. This compares favorably to the iCBT studies for health anxiety were patients averagely completed between 57 and 79% of their treatment modules (Hedman et al., 2011, 2016; Newby et al., 2018). Poor adherence is a widespread challenge, which may limit the potential effectiveness of internet-delivered treatments (Donkin et al., 2011). The particular focus on personal values in ACT and creating a meaningful life may be a possible strength of this treatment model motivating patients to stay in treatment. Accordingly, iACT seems to be a promising new treatment option for health anxiety with some potential advantages over iCBT when it comes to adherence. It may be as effective as iCBT, but more studies are needed; preferably directly comparing iACT to iCBT.

### Adverse events

Adverse events are prevalent in psychotherapy but seldom reported (Rozental et al., 2017). In our trial, only patients in iFORUM (11.6%) reported symptom deterioration at 6-MFU. A meta-analysis of 29 iCBT studies found an average individual deterioration rate of 17.4% in control groups and 5.8% in active treatment groups (Rozental et al., 2017). The lesser deterioration in our control patients may be explained by iFORUM being an active control condition, and some patients did gain a small effect after participation. Still, no patients in iACT reported any deterioration which compares favorably to the reported 5.8% of patients in iCBT.

Regarding negative effects during iACT treatment, the most frequently reported factor was 'symptoms' among the six factors encompassing symptoms, hopelessness, failure, stigma, dependency, and quality (Rozental et al., 2016). Specifically, increased anxiety was reported by 36% of the patients in iACT (item 3). Even though negative effects have not been thoroughly examined in the previous trials, two of the iCBT trials for health anxiety assessed negative effects using an open-ended one-item question (Hedman et al., 2014, 2016). They also found increased anxiety as the main patient-reported negative effect. Consequently, it seems common that patients experience more anxiety during treatment while changing maladaptive behaviors. In our study, few negative effects were reported on dependency of the treatment or clinician, feelings of failure, and hopelessness. Interestingly, only 4% of patients in iACT reported fear of being perceived negatively for undergoing treatment, even though stigma related to psychiatric treatment is a common problem (Rusch et al., 2005). Internet-delivered treatment may inflict less stigma than face-to-face treatment and thus minimize a common barrier to treatment.

### Strengths and limitations

Core strengths of the present study were: randomized design with an active comparison, thorough diagnostic assessment by trained clinicians, well-validated outcome measures, high level of adherence, and low attrition rates. Limitations included lack of post-treatment clinician-based diagnostic assessment, non-blinded allocation for the patients, and treatment crossover which may have confounded the results of iFORUM by decreasing the patients' expectations to its potential benefit. Still, most patients in iFORUM reported small improvements suggesting that it was an active and beneficial control condition. Lastly, applying self-referral could affect the generalizability of the study sample and results. However, in a secondary analysis, we found only minor demographic and clinical differences among self-referred patients in iACT and clinician-referred patients in ACT-G suggesting generalizability of our study sample (Hoffmann, Rask, Hedman-Lagerlof, Eilenberg, & Frostholm, 2019). Altogether, these limitations are not likely to greatly affect the significant results of this study.

### Clinical implications

Patients with health anxiety have often been considered treatment-resistant. There is now substantial evidence for efficacious treatments, and we found that iACT is a new effective, feasible, and easily accessible treatment with minimal adverse effects. The beneficial effects of iACT were obtained without the clinician ever meeting the patient face-to-face during referral, assessment, and treatment. This may have substantial clinical value since harnessing these new technologies has the potential of providing evidence-based treatment to many patients, which is often limited due to capacity, geographical distance, time, and perceived stigmatization. Moreover, the high degree of flexibility reduces inference with patients' daily life, and it might have incremental economic benefits such as the patient not having to take time off work. Harnessing technology such as patient self-referral and internet-delivered treatment may have broad application in the dissemination of health care services. In future studies, we plan to investigate specific mechanisms of change to better understand ‘how’ and ‘why’ iACT translates into the events leading to a successful outcome.

## Conclusions

In Conclusion, patient self-referral and 12 weeks of clinician-guided iACT was an effective and acceptable treatment setup that improved health anxiety and overall mental health. The study contributes to the development of feasible, easily accessible treatment options for patients with health anxiety who often risk being undetected and untreated.

## Supplementary material

For supplementary material accompanying this paper visit https://doi.org/10.1017/S0033291720001312. click here to view supplementary material
