# Supplementary material for: Efficacy of internet-delivered acceptance and commitment therapy for severe health anxiety: results from a randomized, controlled trial
Source: Psychol Med. 2020 May 14;51(15):2685–95. doi: 10.1017/S0033291720001312 (PMC8579157; doi:10.1017/S0033291720001312)
Supplement: Supplementary file 1 [file S0033291720001312sup.zip › S0033291720001312sup001.docx]

| **Questionnaire** | **What is assessed** | **Time points (months)**^a^ |
| --- | --- | --- |
| Whiteley Index-7 (WI-7) | Health anxiety symptoms | Baseline, 1, 2, 3, 4, 10 |
| Health anxiety inventory, Short-form (SHAI) | Health anxiety and negative consequences, two components respectively | Baseline, 4, 10 |
| Symptom Checklist Scale (SCL-92) |  |  |
| Depression subscale (SCL-dep) | General depressive symptoms | Baseline, 4, 10 |
| Anxiety subscale (SCL-anx) | General anxiety symptoms | Baseline, 4, 10 |
| Somatization subscale (SCL-som) | Common physical symptoms e.g., headaches | Baseline, 4, 10 |
| WHO-five Well-being Index (WHO-5) | Quality of life and emotional functioning related to positive mood, vitality, and general interests. | Baseline, 1, 2, 3, 4, 10 |
| Acceptance and Action questionnaire (AAQ-II) | Psychological flexibility | Baseline, 2, 3, 4, 10 |
|  |  |  |
| Negative Effects Questionnaire (NEQ) | Negative incidents and effects of psychological treatment | 4 |

**Supplementary material: Questionnaires**

^a^ 1=randomization. 2=four weeks into treatment. 3=eight weeks into treatment. 4=post-treatment. 10=six-month follow-up
