# Supplementary material for: Efficacy of internet-delivered acceptance and commitment therapy for severe health anxiety: results from a randomized, controlled trial
Source: Psychol Med. 2020 May 14;51(15):2685–95. doi: 10.1017/S0033291720001312 (PMC8579157; doi:10.1017/S0033291720001312)
Supplement: Supplementary file 1 [file S0033291720001312sup.zip › S0033291720001312sup002.docx]

**Supplementary material. Treatment adherence**

|  | **iACT**  (n=53) | **iFORUM**  (n=48) |
| --- | --- | --- |
| **Dropout, n(%)** | 0 (0) | 2 (4) |
| **Treatment completion, n(%)** |  |  |
| Module 1 | 53 (100) | 30 (63) |
| Module 2 | 53 (100) | 28 (58) |
| Module 3 | 53 (100) | 22 (46) |
| Module 4 | 52 (98) | 20 (42) |
| Module 5 | 47 (89) | 20 (42) |
| Module 6 | 38 (72) | 16 (33) |
| Module 7 | 30 (57) | 13 (27) |
| Median (IQR) | 7 (5 - 7) | 2 (0 - 7) |
| **Login, n** |  |  |
| Mean (SD) | 53.8 (22.1) | 14.6 (7.8) |
| Range | 15 - 110 | 3 - 37 |
| **Patient messages/postings, n** |  |  |
| Median (IQR) | 14 (10 - 20) | 2.5 (0 - 6.5) |
| Range | 3 - 44 | 0 - 13 |
| Sent at least one message | 53 (100) | 30 (63) |
| **Clinician messages, n** |  |  |
| Mean (SD) | 23.8 (5.71) | NA |
| Range | 11 – 36 | NA |
| **Worksheets, n** |  |  |
| Mean (SD) | 32.6 (9.43) | NA |
| Range | 10 - 51 | NA |

iACT=internet-delivered Acceptance and Commitment Therapy. iFORUM=internet-delivered discussion forum. n=Number of participants. IQR=interquartile range. SD=Standard deviation.
